# Heart failure with preserved ejection fraction and atrial fibrillation: clinical management in the context of recent therapeutic advances in heart failure and electrophysiology

**DOI:** 10.3389/fcvm.2024.1349584

**Published:** 2024-01-29

**Authors:** Eunice Yang, Haroon Rashid

**Affiliations:** ^1^ Inova Schar Heart and Vascular, Arrhythmia Division, Fairfax, VA, United States; ^2^ Division of Cardiology, Johns Hopkins School of Medicine, Baltimore, MD, United States; ^3^ Virginia Heart, Cardiac Electrophysiology, Falls Church, VA, United States

**Keywords:** atrial fibrillaiton, HFpEF—heart failure with preserved ejection fraction, rhythm control, heart failure, catheter ablation

## Abstract

Heart failure with preserved ejection fraction (HFpEF) and atrial fibrillation (AF) have emerged as major age-related epidemics within cardiology. Both conditions carry overlapping symptomatology, and delineating between AF and HFpEF from a diagnostic standpoint is challenging as echocardiographic and biomarker assessments used to diagnose HFpEF may be impacted by AF. Indeed, these two conditions are commonly found in the same individual, so much so that AF has been used in proposed diagnostic criteria for HFpEF. The frequent concomitant presence of these two conditions is associated with poorer quality of life, exertional capacity, as well as increased risk for decompensated heart failure and all-cause mortality. Though these deleterious effects of AF in HFpEF patients are well described, we currently have only a superficial understanding of the complex interplay between these two conditions. Preliminary studies on intervening in AF in HFpEF are very small, with mixed data on whether modifying the natural history of AF can lead to improvement in heart failure (HF) outcomes in HFpEF. In this review, we will describe the clinical implications of carrying both cardiovascular conditions, address recent advances in HFpEF and AF, and highlight preliminary studies targeted at reduction of effects associated with AF burden in HFpEF.

## Introduction

Heart failure with preserved ejection fraction (HFpEF) and atrial fibrillation (AF) have emerged as major age-related epidemics within cardiology (1, 2). Both conditions carry overlapping symptomatology, and delineating between AF and HFpEF from a diagnostic standpoint is challenging as echocardiographic and biomarker assessments used to diagnose HFpEF may be impacted by AF (3). Indeed, these two conditions are commonly found in the same individual, so much so that AF has been used in proposed diagnostic criteria for HFpEF (4). The frequent concomitant presence of these two conditions is associated with poorer quality of life, exertional capacity, as well as increased risk for decompensated heart failure and all-cause mortality (5–11). Though these deleterious effects of AF in HFpEF patients are well described, we currently have only a superficial understanding of the complex interplay between these two conditions. Preliminary studies on intervening in AF in HFpEF are very small, with mixed data on whether modifying the natural history of AF can lead to improvement in heart failure (HF) outcomes in HFpEF. In this review, we will describe the clinical implications of carrying both cardiovascular conditions, address recent advances in HFpEF and AF, and highlight preliminary studies targeted at reduction of effects associated with AF burden in HFpEF.

### Epidemiology and clinical significance of AF and HFpEF

By 2030, an estimated 12 million Americans will have HF, and at least half of these individuals will have HFpEF (12). HFpEF is a HF condition that leads to HF symptoms similar to individuals with heart failure with reduced ejection fraction (HFrEF) (13). HFpEF patients demonstrate impaired exercise capacity and have poorer projected survival: the median five-year survival rate of HFpEF patients after their first HF hospitalization is 35% (14, 15). At present, there have been limited prospective studies identifying effective treatments that modify the natural course of this disease, with sodium-glucose cotransporter 2 (SGLT2) inhibitors recently identified as the first and only class of drugs offering clinical benefit (16), making HFpEF a major cause of morbidity and mortality with unmet healthcare need.

AF is also a growing cardiovascular epidemic affecting millions of patients worldwide (17, 18). Individuals with AF have increased risk for morbidity and mortality, with a five-fold higher risk for developing HF and cerebrovascular events (15–21). In the general population, the presence of AF, defined in the traditional binary fashion, is independently associated with faster decline in cognitive function with age, a 1.4-fold increased risk of dementia, and a 5-fold increased risk of stroke (7, 22). Failure to diagnose AF and initiate systemic anticoagulation in a timely fashion places these patients at undue risk for cerebrovascular events. Clinical management of AF includes two basic goals: (1) prevention of thromboembolism with systemic anticoagulation when appropriate, and (2) selection of medications and/or interventional procedures to either maintain appropriate heart rate or maintain sinus rhythm.

### AF and HFpEF: proposed interactions and implications on clinical prognosis

The association between AF and HF has been well described, with modern HF cohorts reporting concomitant AF diagnoses in 13%–27% of all HF subjects (23–27). In prospective follow-up of Framingham Heart Study participants, 1,470 participants developed either new AF or HF between 1948 and 1995, with 26% of those participants developing both of these cardiovascular conditions (7). The prevalence of AF in patients with HF is correlated with the severity of the HF condition, ranging from just 5% in patients with mild HF to up to 50% in patients with end-stage HF symptoms (28). Large cohort studies reveal that least one-third of all HFpEF patients have AF, which has been associated with significantly reduced exercise tolerance, increased risk for decompensated HF requiring hospitalization, and overall poorer survival (5–10).

The natural history of AF is for progression from paroxysmal to persistent, and ultimately permanent AF (29). While the adverse cardiovascular events have generally been explored using a binary approach of presence or absence of AF, studies on the general population suggest that the rates of death, stroke and worsening HF are higher in individuals with persistent and permanent AF than in those with paroxysmal AF (30). Interestingly, the presence of any type of AF burden, even paroxysmal AF, appears to increase risk for poor clinical outcomes in HF patients, including patients with HFpEF (29, 30).

### Pathophysiologic interplay between HFpEF and AF

**Figure 1:**
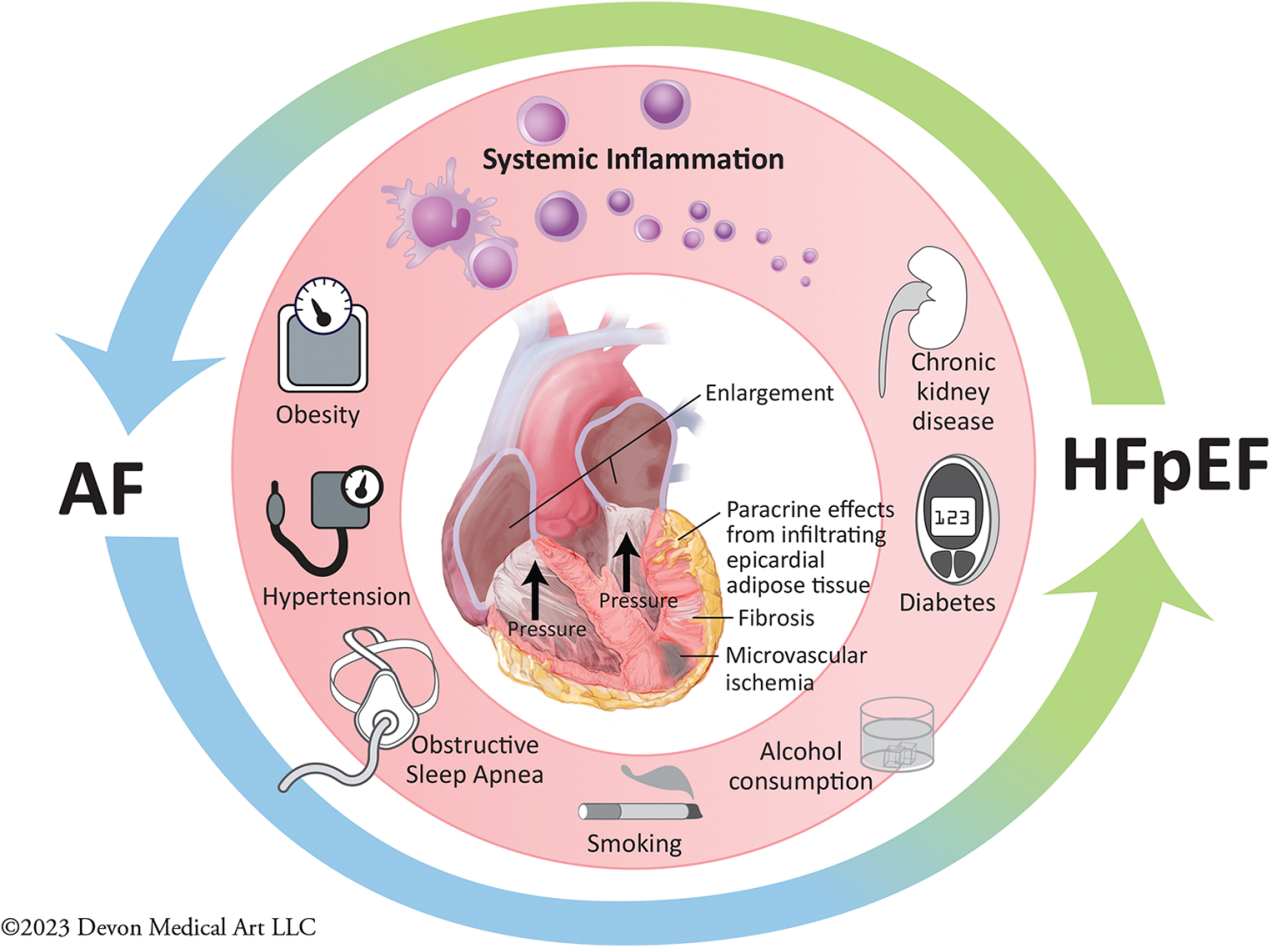
Pathogenesis and progression of atrial fibrillation and heart failure with preserved ejection fraction.

The presence of AF has been associated with increased right and left-sided atrial and ventricular pressures on right heart catheterization (31). Pathophysiologic postulates on the deleterious interplay between AF and HFpEF include these higher right- and left-sided atrial pressures seen in HFpEF with concomitant AF, which may lead to reduced tolerance for fluctuations in intravascular volume, resulting in reduced exercise capacity and increased risk for HF exacerbation requiring hospitalization. This postulate has led to trials assessing the interventional procedures involving placement of an interatrial shunt with the hopes of improving HF outcomes, which have not proven to demonstrate significant benefit (32). However, as shown in Figure 1, the proposed interaction between AF and HFpEF extends well beyond hemodynamic effects alone: patients with both cardiovascular conditions frequent carry other clinical comorbidities such as obesity, hypertension, diabetes, chronic kidney disease, obstructive sleep apnea, alcohol consumption and smoking. These comorbidities all contribute to systemic inflammation, and are associated with higher levels of pro-inflammatory mediators, and longitudinal observational studies report that individuals with higher levels of pro-inflammatory markers at baseline are at higher risk of developing HFpEF and AF during follow-up (33–36). Coronary microvascular dysfunction, defined as myocardial ischemia in the absence of macrovascular epicardial coronary artery disease, has also been shown to be highly prevalent in HFpEF and AF patients, and can cause subtle aberrations in systolic function despite the presence of normal ejection fraction (37). One study assessing prevalence of coronary microvascular dysfunction in HFpEF reported that the prevalence of AF was over twofold higher in HFpEF patients with coronary microvascular dysfunction (58%) than in those without microvascular dysfunction (25%) (38). Other postulated contributors include deposition of epicardial adipose tissue leading to both myocardial infiltration and paracrine effects promoting local inflammation tissue fibrosis and cardiomyocyte dysfunction (39–42), as well as a pro-fibrotic milieu contributing to the pathogenesis and progression of both conditions.

While carrying a concomitant diagnosis of AF portends poorer clinical outcomes in HFpEF, and though many treatments exist to manage AF, no evidence-based treatment guidelines exist for the growing number of patients with HFpEF and AF. Thus, understanding the interplay between AF and HFpEF is vital to guide selection of appropriate therapeutic interventions targeting HFpEF as well as AF to optimize the clinical trajectory of these patients.

### Timing of AF and HFpEF in patients with both disorders: prognostic significance?

Delineating relative timing of diagnosis of AF and HFpEF is particularly helpful as the prognosis of these particular subgroups may differ in response to rhythm control therapy. Amongst these groups are (1) individuals with pre-existing AF and subsequent development of HF; and (2) individuals who have had pre-existing HF before development of clinical AF. The first group with pre-existing AF includes individuals who develop HFrEF with transient or curable etiologies, such as Takotsubo or timely revascularization in ischemic cardiomyopathy, as well as individuals who have HFrEF who have ejection fraction recovery following initiation on goal-directed medial therapy (3, 43). This group also includes individuals with completely reversible ejection fraction after resolution of tachyarrhythmias, for which AF is the most common culprit. In general, the clinical trajectory of individuals diagnosed with AF before developing heart failure who undergo AF control is much more favorable with a higher likelihood of achieving complete recovery from HF symptoms. In contrast, though individuals from the second group who develop AF after already carrying a diagnosis of HF (whether this is HFpEF or HFrEF) may experience clinical benefit in achieving AF rhythm control, these patients typically have poorer long-term clinical projections, including higher thromboembolic risk and increased all-cause mortality (44).

### Novel HFpEF treatments: efficacy for HFpEF patients with AF, as well as effect on risk for development of incident AF

Until recently, extrapolation of medical therapies showing mortality benefit in individuals with systolic HF to HFpEF proved disappointing. Recently, the application of sodium-glucose cotransporter 2 (SGLT2) inhibitors, developed initially for treatment of type 2 diabetes mellitus, have shown major clinical benefits in all HF patients irrespective of diabetes status, including patients with HFpEF (16). The EMPEROR-Preserved and the DELIVER clinical trials evaluated patients with HF with ejection fraction greater than 40%, showing reduction in risk for HF exacerbation, defined as hospitalization or unexpected HF outpatient visit or cardiovascular death (45, 46). The salutary effects of SGLT2 inhibitors was consistent irrespective of whether these patients carried a diagnosis of AF at the time of enrollment, with no reported statistical heterogeneity between the effects of empagliflozin and dapagliflozin. A meta-analysis of these studies demonstrated that the treatment effect noted for the composite endpoint of cardiovascular death or first hospitalization for patients with HFpEF was indeed consistent for patients with AF (HR 0.77, 95% CI 0.69–0.87) and those with no AF (HR 0.83, 95% CI 0.72–0.95), and there was no statistical heterogeneity between empagliflozin and dapagliflozin in the subgroups of patients with AF (47). Analysis of subgroups of patients within the study participants who were at risk for development of AF indicated consistent benefit with SGLT2 inhibitors with no apparent heterogeneity between empagliflozin and dapagliflozin. Dapagliflozin was the only study drug that demonstrated a reduction in incident AF diagnosis compared to placebo, but it was unclear whether this was truly an effect from the drug or a side-effect of improved severity of HF coupled with a higher participant number lending relatively stronger power to identify differences in risk for development of AF following assignment to the treatment arm (48).

Other promising therapies are on the horizon for certain subgroups within the HFpEF population. The STEP-HFpEF trial demonstrated that high-dose weekly administration of semaglutide, a GLP1 receptor agonist, led not only to significant weight loss in obese HFpEF patients, but also resulted in substantial improvement in exertional capacity and reduction in HF symptoms (49). Obesity is a well-described risk factor for AF, and aggressive weight reduction with intensive lifestyle modification has been shown to decrease AF in large randomized control trials (50, 51), so reduction in AF burden should be expected to be seen in obese HFpEF patients who also have AF. Another interesting prospective study demonstrated that HFpEF patients with a permanent pacemaker had significantly improved quality of life, lower N-terminal pro-brain natriuretic peptide levels, and reduced AF burden when their lower rate limit was programmed higher than their underlying resting sinus heart rate, compared against the standard lower rate limit programmatic setting of 60 beats per minute (52). Interplay between these emerging device and medical interventions on AF within the HFpEF population requires further investigation, which is presently under way.

### Modern AF management and implications on HF outcomes in HFpEF

Guidelines for the selection of rate vs. rhythm control strategies has relied on several historical clinical trials, the largest of which was the AFFIRM trial published in 2002 (53–55). Meta-analysis of these studies has not revealed significant differences between pharmacologic rate and rhythm control strategies in risk for all-cause mortality or cerebrovascular events (56). Analysis of the AFFIRM trial, however, did show better outcomes in subjects able to maintain sinus rhythm (53). Following AFFIRM, pharmacologic and procedural advances have led to tremendous advances in our ability to attain rhythm control, with new medications and new therapeutic strategies designed to achieved rhythm control. A central discovery was the identification of ectopic beats originating from the pulmonary veins as the major triggers for initiation of AF has led to an ablation strategy for AF rhythm control that involves pulmonary vein isolation (PVI) (57). AF ablation incorporating PVI, now carries a class I indication for treatment of individuals with symptomatic paroxysmal AF that is refractory to at least one anti-arrhythmic medication, and a class IIa recommendation for individuals with recurrent paroxysmal AF even before therapeutic trials of antiarrhythmic drug therapies (58). Other shifts in AF management noted in comparing AFFIRM with the recently published EAST-AFNET4 study show decreased in use of digoxin, increased availability of newer anti-arrhythmic options, such as dronedarone (59, 60).

Recent studies comparing these current rhythm control strategies to rate control strategies have shown more clinical benefit of pursuit of rhythm control both in the general population as well as in patients with HF, in contrast to earlier landmark trial findings (61–63). As recent studies have highlighted the enhanced likelihood for success of achieving rhythm control with early intervention, pursuit of early intervention on AF in the HFpEF population may mitigate the effects of this rhythm in this population.

The evidence supporting AF ablation that incorporates PVI for AF management in the HFrEF population has grown tremendously over the last 10 years. Following an initial observational study that showed that patients HFrEF and AF tended to perform better after undergoing successful electrical cardioversion (64), a series of small studies were conducted comparing AF ablation with medical management in the HFrEF population—all of which demonstrated significant improvement in ejection fraction and exercise capacity (61, 65, 66). Multicenter randomized control trials following these studies all demonstrated the same improvement in cardiac function as well as small but significant reduction in mortality (63, 67). The mechanism driving improved clinical outcomes in these studies remains unknown, as does the utility of this intervention in the HFpEF population, given that most therapies showing improved outcomes in HFrEF have not demonstrated comparable benefit in HFpEF.

Preliminary single-center exploratory studies have been published describing benefit of AF rhythm control using a catheter ablation strategy compared to a rate control strategy, including ones that demonstrate a reduction of pulmonary capillary wedge measurements following AF ablation in HFpEF patients (68, 69). To our knowledge, no large scale prospective randomized controlled trials have yet evaluated whether AF ablation improves clinical outcomes in the HFpEF population. Pre-specified subgroup analysis of the EAST-AFNET4 study compared early rhythm control of AF and found that it was associated with a lower risk of adverse cardiovascular outcomes in comparison with “usual care” among patients with AF diagnosed within 1 year of study enrollment, which included patients with HF (*n* = 798), approximately half of whom had HFpEF (56%) (70). The primary outcome of the EAST-AFNET4 study was a composite outcome (including death from cardiovascular causes, stroke, HF hospitalization or hospitalization secondary to acute coronary syndrome) which occurred in 94 of 396 (24%) HF patients assigned to early rhythm control and in 130 of 402 (32%) HF patients randomly assigned to usual care [hazard ratio (HR) 0.74, 95% CI 0.56–0.97, *p* = 0.03]. As reported in the subgroup analysis, patients with HFpEF demonstrated more improvement in reported NYHA class than patients with HFrEF, and HFpEF participants appeared to have an overall lower risk for time to development of the primary composite outcome in comparison to their HFrEF counterparts. Finally, exploratory analysis of HFpEF patients suggested that treatment with amiodarone, but not treatment with flecainide, propafenone or dronedarone, was associated with early HF hospitalizations (70). This association may be due to patients receiving amiodarone having a higher frequency and severity of clinical comorbidities serving as contraindications for other anti-arrhythmic medications, but serves to temper our complacency in using amiodarone as a rhythm control treatment option in the HF population. Long-term use of amiodarone may require re-examination, particularly in the face of the other rhythm control options available in our current armamentarium of AF therapies in HF patients.

The majority of EAST-AFNET4 patients were prescribed anti-arrhythmic drugs as the first line early rhythm control option, with only a small fraction of these patients undergoing AF ablation. A post-hoc analysis of the CABANA clinical trial compared catheter ablation and antiarrhythmic drug therapy in 778 patients with AF and stable HF at baseline, the vast majority (79%) of whom had HFpEF (71). In this secondary analysis, catheter ablation was associated with a striking 36% relative reduction in the primary composite endpoint of death, disabling stroke, serious bleeding, or cardiac arrest and a 43% relative reduction in all-cause mortality compared to anti-arrhythmic drug therapy. Notably, there was no significant reduction in the frequency of HF hospitalizations and there were limited data to ascertain HFpEF diagnosis. The authors concluded that these results should be reproduced in a confirmatory study dedicated to looking at the HFpEF population.

It should be noted that patients with HFpEF often present with permanent AF with profound left atrial remodeling. These patients may not have a high likelihood of maintaining sinus rhythm despite attempts at rhythm control using catheter ablation and/or antiarrhythmic drug therapy. When ablation and anti-arrhythmic drug strategies can no longer achieve rhythm control, atrioventricular nodal ablation with placement of a biventricular or conduction system pacemaker should be discussed in HFpEF, as the APAF-CRT trial for those with permanent AF and narrow QRS hospitalized for HF demonstrated reduction in all-cause mortality, irrespective of ejection fraction (72).

## Conclusion

While the treatments available for HFpEF and AF have improved significantly within the last few years, patients who carry both clinical diagnoses are still faced with limited evidence guiding their clinical management. This unmet need is an opportunity for investigation and improvement of clinical outcomes, and requires not only the cooperation of the HF and electrophysiology teams, but requires a multidisciplinary approach to treatment to target comorbidities and lifestyle modifications (73, 74). We firmly believe that implementation of cross-disciplinary management is essential to success of managing all AF patients and carries the greatest potential for benefit in this patient subgroup.
